# Impact of Microscopic Motility on the Swimming Behavior of Parasites: Straighter Trypanosomes are More Directional

**DOI:** 10.1371/journal.pcbi.1002058

**Published:** 2011-06-16

**Authors:** Sravanti Uppaluri, Jan Nagler, Eric Stellamanns, Niko Heddergott, Stephan Herminghaus, Markus Engstler, Thomas Pfohl

**Affiliations:** 1 Max-Planck-Institute for Dynamics and Self-Organization, Göttingen, Germany; 2 Institute for Nonlinear Dynamics, Faculty of Physics, Georg-August-Universität, Göttingen, Germany; 3 Biozentrum, Department for Cell and Developmental Biology, University of Würzburg, Würzburg, Germany; 4 Department of Chemistry, University of Basel, Basel, Switzerland; Institute Pasteur & CNRS, France

## Abstract

Microorganisms, particularly parasites, have developed sophisticated swimming mechanisms to cope with a varied range of environments. African Trypanosomes, causative agents of fatal illness in humans and animals, use an insect vector (the Tsetse fly) to infect mammals, involving many developmental changes in which cell motility is of prime importance. Our studies reveal that differences in cell body shape are correlated with a diverse range of cell behaviors contributing to the directional motion of the cell. Straighter cells swim more directionally while cells that exhibit little net displacement appear to be more bent. Initiation of cell division, beginning with the emergence of a second flagellum at the base, correlates to directional persistence. Cell trajectory and rapid body fluctuation correlation analysis uncovers two characteristic relaxation times: a short relaxation time due to strong body distortions in the range of 20 to 80 ms and a longer time associated with the persistence in average swimming direction in the order of 15 seconds. Different motility modes, possibly resulting from varying body stiffness, could be of consequence for host invasion during distinct infective stages.

## Author Summary

Single cell motility is essential for many physiological functions. We seek to further our understanding of the swimming mechanism of trypanosomes - parasites responsible for deadly disease in humans and cattle. Trypanosomes are found both in the blood stream and invading tissue spaces. The swimming mechanisms used by microorganisms, such as bacteria and parasites, are very different from those used in our macro-scale environment. The trypanosome's ability to swim, mediated through a flexible rod like structure called a flagellum, attached to the length of the body, is essential for its survival. We found that cell shape (or elongation) correlates to directionality in cell movement. Additionally, using high-speed microscopy we uncovered extremely fast dynamics of the flagellum tip, up to 50 times faster than the whole cell swimming speed. Together our results imply that physical parameters such as cell stiffness may influence a trypanosome's ability to invade tissue.

## Introduction

Eukaryotic cell motility plays a key role in many physiological functions including cell division, development, survival, as well as disease pathogenesis [1]–[4]. Studies of single cell motility have not only uncovered different aspects of these functions, but also provide great biophysical insight into life at low Reynolds numbers [5]–[8]. Trypanosomes, found across Africa and South America, are causative agents of diseases that are endemic in low income areas [9]. Recent work demonstrating that cell surface hydrodynamic drag is used by trypanosomes to sweep antibodies to the flagellar pocket, the ‘cell mouth’, for host immune evasion, has piqued interest in trypanosome motility [10], [11]. In this study, we examine the microscopic swimming behavior of the model organism *Trypanosoma brucei brucei*, the pathogen responsible for animal African trypanosomiasis.

**Figure 1:**
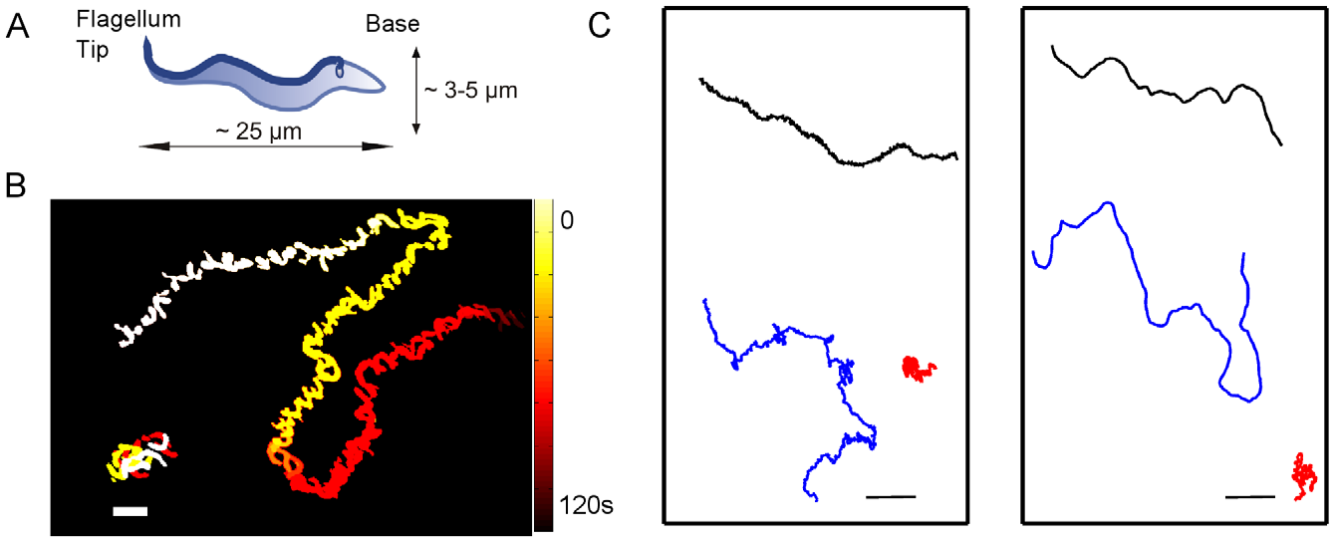
Trypanosome trajectories. a) Scheme of a typical trypanosome cell shape with dimensions. The flagellum originates at the flagellar pocket, near the kinetoplast and runs along the entire body b) Time-lapse overlay of trypanosomes trajectory illustrates tumbling (lower left), and running motion (upper right), scale bar 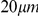, colorbar represents time, both trajectories represent about 2 minutes of trypanosome swimming. c) Diversity in trypanosome trajectories, reveals three motility modes in which cells tumble (tumbler, in red), travel directionally (persistent, in black), or alternate between tumbling and running motion (intermediate, in blue). Left panel derived from experiment, right panel from model, scale bars 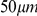.

As in other motile cells such as *E.coli* and spermatozoa, *T. brucei brucei* motility is mediated by a flagellum. However, unlike sperm and *E.coli*, the flagellum of the trypanosome emerges from the flagellar pocket near the base of the cell and runs along the length of the entire body as illustrated in Fig. 1 . Until very recently it was believed that the flagellar beat originates at the tip and is carried to the base of the cell resulting in a corkscrew like forward movement [12]–[14]. Branche *et al.* showed that base to tip wave propagation resulted in reorientation of the whole cell without any significant backward movement [14]. Work by Rodriguez et al. suggests that both bloodstream form and procyclic form cells move by anti-chiral helices separated by a kink traveling along the length of the body during wave propagation [15]. RNAi-based ablation of flagellar proteins in bloodstream-form parasites has resulted in loss of cell viability demonstrating the importance of cell motility and in particular the flagellum in cell survival [3], [16]. The discovery of social motility in trypanosomes [17] further motivates a deeper look into their motility.

While there have been many efforts to uncover the molecular biology of motility in trypanosomes and other microorganisms, a quantitative understanding of parasite motility is still lacking. However, recent temporal correlation analysis of experimentally derived trajectories suggests the presence of two characteristic relaxation times in trypanosome motility which could be used to construct a model of Langevin equations to describe trypanosome motility. A short relaxation time is attributed to the strong body distortions that the cell undergoes during swimming, while a significantly longer relaxation time is associated with the persistence in average swimming direction (submitted, Zaburdaev V. et al.). All studies of trypanosome motility have focused exclusively on cells swimming directionally [12], [15]. However, it is known that more than half of a trypanosome population is dividing under normal culture conditions underscoring the importance of carrying out population-wide analysis for quantitative characterization.

## Results/Discussion

In this work, we aim not only to characterize differences seen in swimming behavior within a single population, but also to link these behaviors to the microscopic physical processes that may explain these observations. To this end, we examined motility of single trypanosomes in a quasi two-dimensional environment. A Pearson random walk model [8], [18], [19] is used to describe the swimming trajectories of trypanosomes. We further suggest that the observed diversity in swimming trajectories may be correlated to varying cell stiffness.

### Trypanosome trajectories

Recordings of swimming cells in culture medium were taken at 7 Hz and processed to obtain single cell trajectories which follow the center of mass of the cell. Characteristic time-lapse trajectories are given in Fig. 1 b and corresponding movies are given in the Supplementary Information (Video S1 and Video S2). The cells are in a homogeneous environment with no chemical gradients, therefore specific chemo-attraction may be ruled out as the basis for cell locomotion in this study. It is worth mentioning that although chemotaxis has not been demonstrated in trypanosomes, it is likely that they are capable of it [20].

As seen in the example trajectories of Fig. 1 c, trypanosomes from the same population taken from the same cell culture which are exposed to identical environmental conditions evidently do not follow a single *motility mode*.

About a quarter of the trypanosomes ‘tumble’ with no persistence in direction (shown in red in Fig. 1 c). These cells, referred to as *tumbling walkers* (TW), do not seem to have a well defined orientation, hence the most frequently described flagellum end-led swimming is not discernible.

Secondly, close to half of the swimmers are highly directional (*persistent walkers* - PW) with a complete absence of tumbling motion within the observation interval. Cell orientation of these swimmers remains constant, with the flagellum tip leading in the swimming direction.

The remaining population is comprised of cells that swim directionally with constant cell orientation but occasionally stop, tumble and reorient themselves and then move directionally again. These trajectories resemble those of *E.coli* in which steady forward motion is interrupted by tumbling [8]. We refer to these cells as *intermediate walkers* (IW).

**Figure 2:**
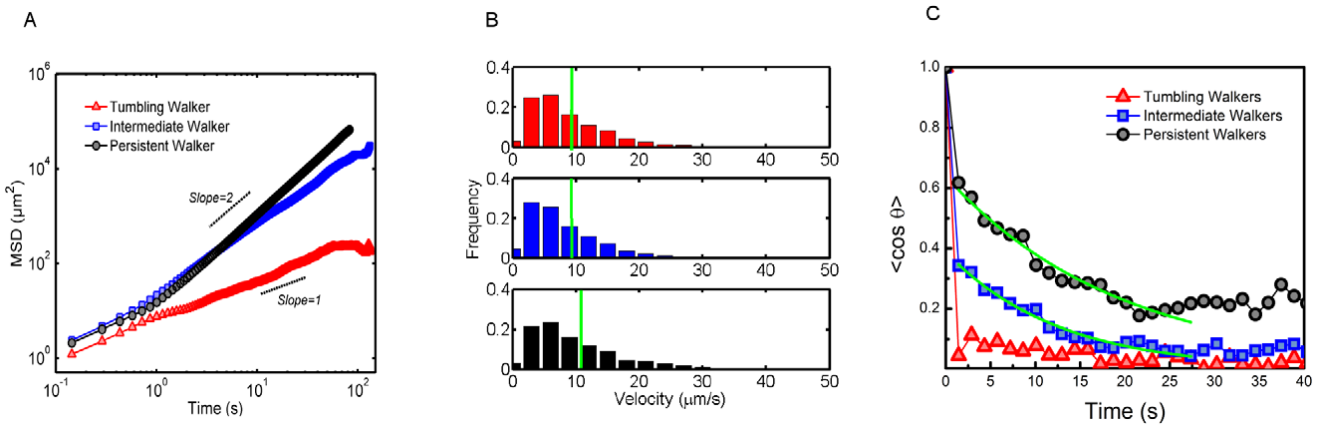
Similarities and diversity of trypanosome motion. (a) Mean squared displacement of typical swimmers for each mode (b) Population wide velocity distributions are virtually identical for all motility modes (derived from with profiling time 1/7 s). Mean velocity is shown in green. (c) Average cosine correlation function for each motility mode, exponential fits for persistence time shown in green.

Cell swimming was characterized using mean squared displacement (MSD) given by 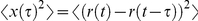, where 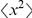 is the MSD, *r* is position, and 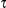 is the time interval - bound only by the time resolution of the experiment - see Fig. 2 a. The scaling exponent 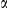, with 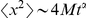 where 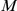 is the motility coefficient [21], [22], gives a measure of the degree of directional correlation. Tumblers are characterized by an average scaling exponent near 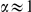, indicating uncorrelated motion. The intermediate walkers, exhibit more correlation and for persistent walkers, 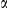 tends to increase well above unity, indicating longer term correlations in swimming direction [21], [23]. See Table 1 for a summary.

**Table 1:** Summary of trypanosome motility modes.

Swimming Mode	Tumbling	Intermediate	Persistent
Average Velocity 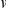 ( 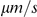 )	8.42	8.90	10.7
Persistence Time, 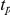 (s)	-	12.4	19.3
Persistence Length, 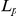 ( 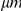 )	-	110.4	206.5
Average Scaling exponent, 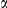 (data)	1.2	1.4	1.6
Scaling exponent, 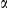 (model)	1.0	1.4	1.5

The average velocity 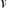 remains fairly constant in all motility modes, however the persistence time, 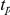 reveals differences in motility modes arising from directional motion. The persistence length is given by 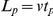. Differences in persistence are also represented in the scaling exponents obtained from power law fits of the MSD.

Interestingly, as shown in the distribution of Fig. 2 b all three types of swimmers have virtually the same average swimming speed, demonstrating that these motility modes arise primarily due to directional motion. Note that here speed is defined by the distance covered by the center of mass from frame to frame of experimental recordings. Thus, a tumbling cell also exhibits a speed, even if there is no net displacement. The differentiation characteristic of the motility modes is the persistent motion.

The time scale at which directional persistence is lost is characterized by the cosine correlation function,
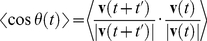
(1)


where **v** is the velocity vector, 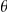 is the angle between the two adjacent vectors separated by the time interval 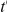, and 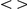 denotes an ensemble average. In the case of TWs, the correlation decays rapidly and remains close to zero as shown in Fig. 2 c. For both persistent walkers and intermediate walkers a rapid decay in correlation is also seen, followed by a second slower decay. Sharp correlation drops for small time lags likely arise from the strong body distortions mentioned previously. An exponential fit to the second slower decay (shown in green in Fig. 2 c) of the average cosine correlation function for persistent walkers reveals a persistence time of 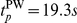. For cells that alternate between tumbling and directional swimming, we find the average perlsistence time significantly smaller, 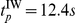. The mean tumbing interval is 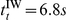 - much longer than the tumble time of 0.1 s seen in *E.coli* [8]. Here a ‘tumbling interval’ was derived by the maximal time for which the distance traveled stays virtually constant with respect to time. Interestingly, all three correlations appear to remain above zero in the observation interval pointing to yet another longer term correlation.

Tumbling walkers for which all turn angles have equal likelihood are characterized by a flat turn angle distribution, while individuals that are directionally persistent draw from a rather narrow turn angle distribution. We use the spread of the turn angle distributions 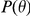 - related to 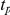, of each trajectory to systematically categorize individuals into their respective swimming modes.

Table 1 summarizes the quantitative differences between the motility modes - highlighting the striking diversity in motility in a single trypanosome population.

**Figure 3:**
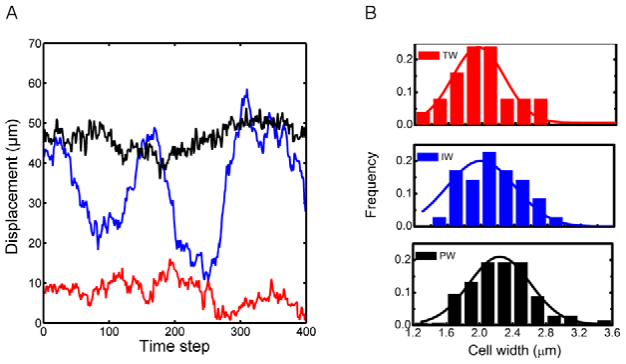
Distinguishing features of the motility modes. (a) Typical displacement calculated between every 100 frames of recordings for each motility mode. While TWs and PWs maintain low and high displacement values respectively, IWs seem to alternate between these two modes. (b) Distribution of cell width (measured at the widest point) for cells of each motility mode.

Fig. 3a illustrates typical displacement calculated between every 100 frames of recordings for each motility mode. It clearly demonstrates that while TWs and PWs maintain low and high displacement values respectively throughout the observation interval, the IWs alternate or switch between the two ‘states’ thus warranting the classification of trypanosome motility into three distinct modes. The physical differences correlated to these motility modes are discussed below.

### Characterizing motility modes

We describe a simulation model with which we are able to reconstruct the motion of the three motility modes. A Pearson random walk model [8], [18], [19] is used where step size and turn angle are determined by two independent distributions, 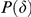 and 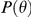 respectively. The Pearson random walk is one of the simplest conceivable models which accounts for stochastic but directional motion. It has been used as a paradigmatic model for locomotion in animals widely used in microorganisms but also in butterflies, for instance. It is therefore a suitable minimal model for the locomotion in trypanosomes. Fortunately, we found that this minimal model describes the locomotion in trypanosomes to a very satisfactory extent. The turn angle is determined by the previous displacement direction and a randomly drawn turn angle. Step sizes are drawn from an exponential distribution 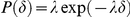 with a characteristic displacement [18] 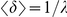 (equivalent for each motility mode) - see Supplementary Information Text S1 for details. The model captures the motion of the trypanosomes, in particular their differences in persistence of translation motion see Fig. 1 d and table 1. However, it does not capture the smaller scale ‘jaggedness’ seen in the trajectories. This jaggedness arises from the rapid bending and twisting of the trypanosome cell body during swimming. To further characterize the dynamics of trypanosome motility at these smaller time scales we utilized high speed microscopy.

### Microscopic origin of motility modes

In order to investigate the physical mechanisms for the observed motility behaviors, we examine trypanosomes at higher magnifications and at a much higher sampling rate of 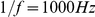 (See Supplementary Information Video S3 and Video S4). Very recent work with high speed microscopy has demonstrated surprisingly fast flagellum tip velocities up to 25 times faster than the average cell swimming speeds [15] further highlighting the need for high temporal resolution towards gaining a physical understanding of this parasite's motility.

The flagellum runs along the cell body resulting in complex body deformations during swimming, and while the quantitative descriptions of flagellar movements have been done for other species [24], little has been been done for *T. brucei brucei*.

**Figure 4:**
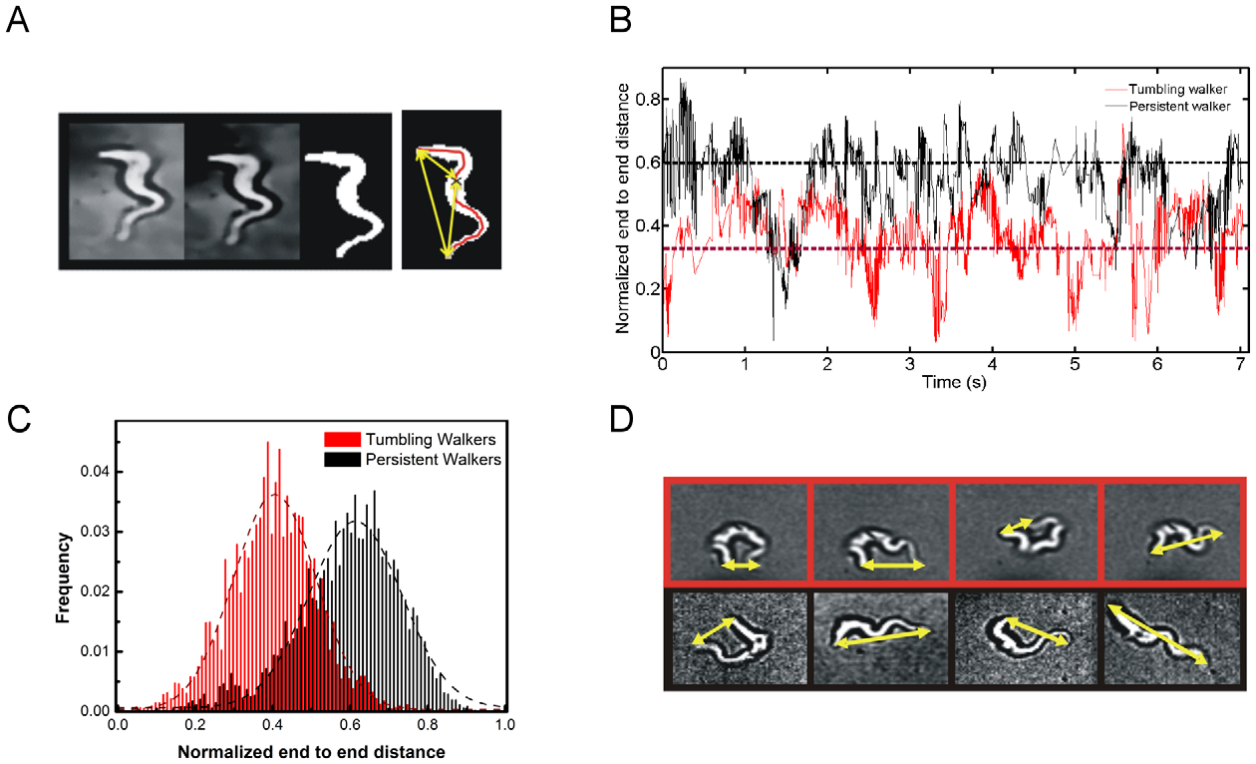
End-to-end distance. (a) Image processing to obtain a skeleton line, and thereby extract the end-to-end distance. (b) Typical time series of end-to-end distance (normalized to cell length) (c) Population distributions of end-to-end distance in persistent and tumbling walkers (d) Snapshots from movies showing that persistent cells are indeed more stretched (black boxes) than tumbling walkers (red boxes).

We chose a straightforward approach and examined the variations in the distance between the two ends of the cell (referred to as end to end distance here on) over time. Trypanosomes were tracked to ascertain their motility mode, and then followed by high speed recordings of their movement at a higher magnification. Recorded images were processed as described in the methods section. A skeleton line through the center of the cell body was obtained as illustrated in Fig. 4 a. The end to end distance, indicated by the yellow arrow in Fig. 4 a was calculated and normalized by the cell length itself (represented by the length of the single pixel line in red). Thus resulting in a time series of the normalized end-to-end distance which allows for comparison of motility modes as shown in Fig. 4 b (see Supplementary Information Videos S3 and S4). Fig. 4 b shows that the persistent cell is consistently more stretched or elongated than the the tumbler.

Histograms of the end-to-end distances [25], [26] show clear differences between the swimming behaviors ( Fig. 4 c). A higher mean end-to-end distance for the PWs, indicates that directional persistence is correlated to an elongated cell shape. Snapshots of the high speed movies of individuals demonstrate, as shown in Fig. 4 d, that cells swimming with directional persistence appear to take a more stretched body shape. Indeed the mean end-to-end distance of persistent cells is 0.6 - more than 1.5 times that of tumblers for which it is 0.38.

We ascribe the shape of the trypanosome to a worm-like chain [25], [27]. The mean squared end-to-end distance 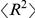 is given by 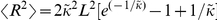 [27], [28]. Where 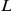 is the length of the chain, and 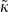 is a dimensionless variable dependent on flexural rigidity, 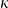, and energy utilization for motility, 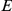. Assuming equal energy utilization for self propelling motion in both motility modes, that is 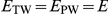, the ratio of the end-to-end distances of the two motility modes is given by
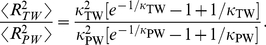
(2)


Based on the above assumption of equal energy utilization, we find that persistent cells have three times more flexural rigidity than tumbling walker cells. The directional cells may be stiffer due to reorganization of motor proteins and crosslinking within the microtubules found both in the cell body and the flagellum. Cell division is associated with the growth of a second flagellum alongside the old one which may also contribute to differences in cell stiffness (see below). The assumption of equal energy utilization for motility from one cell to another is supported but not confirmed by the fact that the velocity distribution for all motility modes is the same. It is not unlikely that these observations are due to an interplay of differences in flexural rigidity and also energy utilization for cell motion which may depend on cell length [29]. The precise differences in flexural rigidity and energy utilization among the motility modes remain to be confirmed through direct measures in further studies.

Nevertheless, these results are in qualitative agreement with theoretical work by Wada and Netz [30] on motility of the bacterium *Spiroplasma* in which softer cells were shown to flex more significantly due to random thermal fluctuations and thus were less efficient in directional motion. *Spiroplasma* has previously been reported to swim using a kinking helical mechanism [31] similar to the one recently suggested by Rodriguez et al. [15] for trypanosome swimming. Further theoretical work suggests that changes in flexural rigidity of the cell have a direct effect on the pitch of the helical movement [32].

Cell division for blood stream form trypanosomes begins with the growth of a new flagellum. The cell body begins to expand while the kinetoplast, attached to the basal body, is replicated. Mitosis of the nucleus begins while the second flagellum continues to grow. Cell width may be used an indicator for the expansion associated with cell division. For this study, while only single cells that appeared to have a single flagellum were selected the exact stage of the cell cycle can only be assessed by measuring the cell width in this experiment. In Fig. 3 b the distribution of cell width (measured at the widest point) for cells in each motility mode is shown. On average, persistent walkers are wider than the other two motility modes, indicating that they are already involved in cell division and the growth of a second flagellum. How cell motility is affected once cytokinesis progresses and daugther cells begin to resolve requires further investigation.

**Figure 5:**
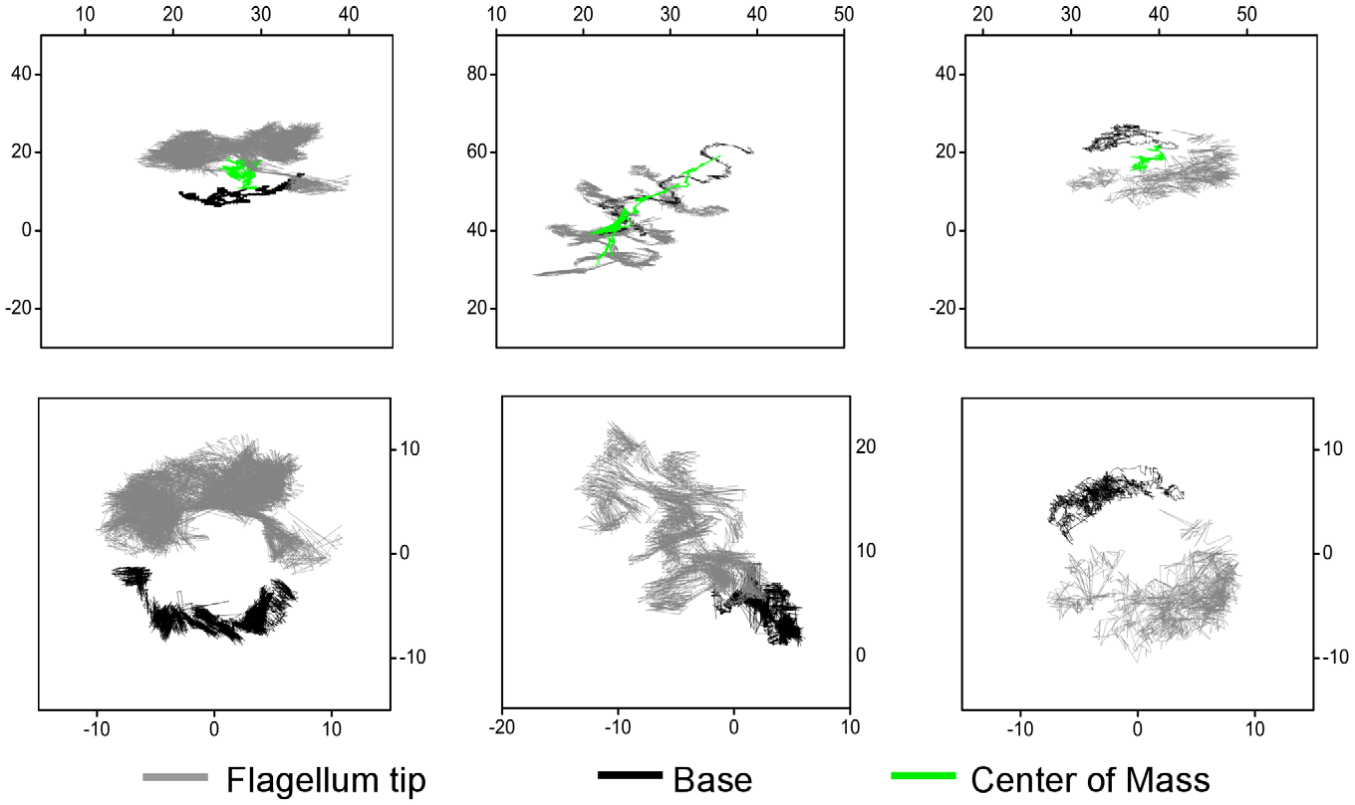
Cell-end trajectories. Trajectories of tip (grey) and base (black) as well as center of mass (green) shown for 9 s (units are 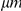). Top three panels show the movement of the flagellum tip and base as well as the center of mass. Lower panels show the corresponding cell-end trajectories with a center of mass correction allowing us to isolate center of mass movements from cell-end movements. Left and right panels: persistent walkers, middle panel: tumbling walker - persistence is not apparent at this time scale.

Aside from giving us clues about the microscopic origin of the observed motility modes, this data also allows us to study the dynamics of cell movement to further our understanding of the swimming mechanism of the trypanosomes. We track the movement of the base and flagellum tip with respect to the center of mass of the cell, allowing us to isolate the whole body movement from the movement of the cell ends alone. Typical trajectories shown in Fig. 5 indicate that the flagellum tip appears to move faster than the base of the cell.

**Figure 6:**
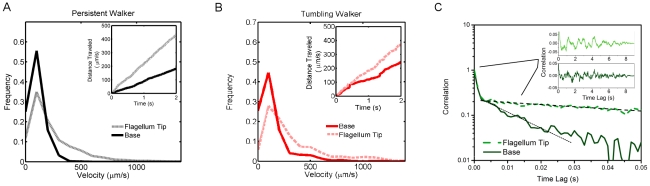
Velocity and correlations in cell-end movements. Typical velocity distributions for (a) Persistent walkers and (b) Tumbling walkers (shown relative to center of mass), insets are distance traveled vs time for typical individuals - the mean slopes were used to calculate cell-end velocities listed in the text. In both swimming modes, the tip moves much faster than the base. (c) Typical velocity autocorrelation of cell ends. Both persistent and tumbling walkers exhibit a faster decay in velocity autocorrelation for the base than the flagellum tip (exponential fit - dashed black line). Inset: linear plot of correlation, shows a periodicity in velocity at 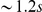.

Velocity distributions of cell ends, extracted from the trajectories of cell ends are shown in Fig. 6 a and b. We obtain mean flagellum tip velocities of 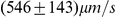 and 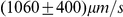 for the tumbling walker and persistent cells respectively while base-end velocities are much lower for both TWs and PWs 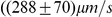 and 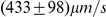 respectively) - all in fair agreement with [15]. This finding points to a ‘velocity gradient’ along the cell body, which is lowest at the base of the cell and increases toward the flagellum end. The gradient, which appears to be steeper in persistent walkers, in turn may stem from a gradual increase in elasticity due to the tapering body shape and from the bias in the hydrodynamic center of mass toward the base of the cell. Changes in overall cell stiffness would hence not only affect flagellar velocities, but also the directionality in cell-end movement, thus determining the motility mode of the cell.

Notably, we find extremely fast cell body dynamics; the flagellum tip of the persistent walker moves over 40 times faster than the average reported whole cell swimming speed of 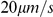. For the tumbling walker, there is a 20-fold difference between the tip and the whole cell speed. Together these results support the notion that the motility modes are a consequence of cell elongation and could be correlated to cell stiffness. The single pixel line to represent the cell body provides a straightforward method for further investigations into the movements of a cell and may shed light on the kinking mechanism recently suggested for trypanosome movement [15].

Finally, using a velocity autocorrelation function [33] for each cell end we are able to extract the fast time scales relevant to the cell movement. The correlation function is given by
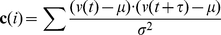
(3)


where velocity 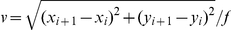, 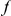 is the temporal resolution, 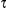 is the profile time or time lag, 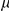 is the mean and 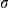 is the velocity standard deviation. Eq. (3) reveals how fast the correlation in velocity is lost (temporal decay) and uncovers any underlying periodicity in velocity. In Fig. 6 c, a typical velocity autocorrelation function is shown for the base and tip of a cell.

An exponential fit to the correlation function reveals that the decay time is 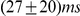 and 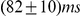 for the base and tip respectively. These decay times do not appear to vary significantly across the three motility modes and point to surprisingly fast dynamics in cellular motility due to the fast body distortions. Note that the first fast decay is attributed mainly to resolution effects and therefore ignored in the fitting. Further a longer periodicity of 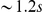 is seen in the autocorrelation functions ( Fig. 6 c inset). This periodicity is likely to arise from the repeated ‘bend and release’ motions of the cell body which can clearly be seen in the trajectory shown in Fig. 5 middle panel.

Although trypanosome run and tumble motion has been reported previously [14], [34], different motility modes in *T. brucei brucei* had never been characterized in detail. Directionality may confer the ability to invade into tissue - the last stage of sleeping sickness is characterized by parasite invasion of the blood brain barrier [35], or it may be an effective nutrient search strategy[36]. On the other hand, cell swimming has been shown to be essential for host antibody removal and one of these motility modes may increase local hydrodynamic drag on surface proteins [10]. Our results indicate that macroscopic motility modes could be correlated to varying cell stiffness. Direct measurements of cell stiffness are required to confirm this hypothesis. The analysis of cell end-to-end distance provides a rapid screen for identification of differences in microscopic properties of cells. Finally our analysis points to remarkably fast cell motility dynamics influencing both the intrinsic rotational and translational cellular motion. Extending the present experiments to include flow for further investigations into the biological relevance of the motility modes may thus help identify the importance of cell swimming in various infective stages.

## Materials and Methods

### Trypanosomes

A mixed population of monomorphic wildtype bloodstream form Trypanosoma brucei brucei, strain Lister 427, clone 221a (MITat 1.2) grown in HMI9 complete medium were cultured at 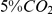, 37°C and harvested at a cell density of 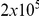 cells/mL. Cells were resuspended in fresh medium before every tracking experiment.

### Microscopy

An Olympus BX61 microscope with 20x and 60x oil objectives equipped with either a PCO SensiCamQE camera, or a Phantom Miro (Vision Research) camera for higher temporal resolution movies were used. During recording the image is taken very slightly out of focus enhancing image contrast. High speed movies were recorded at 1000 frames per second the 60x oil objective, 1.25 aperture, for a minimum of 7 seconds.

### Image Processing

Each movie frame is processed using Matlab's (2009a, The MathWorks) image processing toolbox. The images were processed with gaussian filters and then processed to a black and white image using the appropriate threshold. The in-built skeletonization feature of Matlab was used to obtain a single pixel line representing the center line running through cell body. The end points of the skeleton line are detected and distance between these two points is recorded. Thus, a time series of normalized end-to-end distances is obtained.

### Tracking

Cell swimming was observed between two microscope slides at room temperature in HMI-9 complete medium in a 2-dimensional setting between a microscope slide and a coverslip, cleaned by sonication in isopropanol and dried with high pressure. Cell solution was sufficiently dilute to allow for tracking of isolated individual cells without any contact with neighboring cells. Recording were made within 30 min of removal from the 37°C incubator. Cell trajectories are derived from movies collected at 7 Hz using transmission light with a 20x oil objective using the track object feature of the Image-Pro Plus software from Media Cybernetics.

### Analysis

All statistical analysis was done using MATLAB. All fitting was done using Origin (2008, Origin Lab Corporation) with an exponential decay, 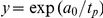.

## Supporting Information

Text S1 Details of Pearson random walk model used to describe trypanosome trajectories.
(PDF)

Video S1 Trajectory of a persistent walker. All movies are displayed at 10 Hz. Samples were taken slightly out of focus to facilitate image processing. Recorded at 7 frames per second and at 20x magnification, background subtraction was done using Image Pro Plus. The track corresponds to the center of mass of the cell.
(WMV)

Video S2 Trajectory of a tumbling walker. All movies are displayed at 10 Hz. Samples were taken slightly out of focus to facilitate image processing. Recorded at 7 frames per second and at 20x magnification, background subtraction was done using Image Pro Plus. The track is not shown for clarity.
(WMV)

Video S3 High speed recording of a persistent walker. Recorded at 1000 frames per second and at 60x magnification.
(WMV)

Video S4 High speed recording of a tumbling walker. Recorded at 1000 frames per second and at 60x magnification.
(WMV)
