# Supplementary material for: Impact of Microscopic Motility on the Swimming Behavior of Parasites: Straighter Trypanosomes are More Directional
Source: PLoS Comput Biol. 2011 Jun 16;7(6):e1002058. doi: 10.1371/journal.pcbi.1002058 (PMC3116898; doi:10.1371/journal.pcbi.1002058)
Supplement: Text S1 — Details of Pearson random walk model used to describe trypanosome trajectories. (PDF) [file pcbi.1002058.s001.pdf]

Text S1

Model

We consider a Pearson random walk [8,17] with randomly drawn exponential distributed displacements δ and turn angles θ. Let

$$P(\delta) = \lambda e^{-\lambda\delta} \tag{1}$$

the probability distribution function (pdf) for the displacements and

$$P(\theta) = c_N \; e^{-\gamma|\theta|} \tag{2}$$

the pdf for the turning angles where $-\pi \leq \theta \leq \pi$; c_N being the normalization constant $c_N = \gamma(1 - e^{-\pi\gamma})$. Each time step $t = 1, 2, \ldots$, the displacement r_t and the turn angle θ_t are chosen. Thereafter the 2D displacement vector is added to the actual position vector,

$$\vec{R}_{t+1} = \vec{R}_t + \vec{r}_t \tag{3}$$

where $\vec{r}_t = r_t \left[\cos\left(\theta_{t-1} + \theta_t\right), \sin\left(\theta_{t-1} + \theta_t\right)\right]$.

It is interesting to note that first, *any* symmetric peaked shape of the turn angle pdf with well defined variance, can serve as a generating pdf for a Pearson random walk.

Second, for any $\gamma < \infty$ the Pearson walk becomes a normal random walk in the limiting case $t \to \infty$. Thus the mean-square displacement (MSD) is asymptotical linear in time, $\langle \vec{R}_t^2 \rangle \sim t$. However, for intermediate time scales $t \approx 1/\gamma$, the Pearson walker exhibits directional, or so-called persistent, motion, being an intermediate regime between normal diffusion $\langle \vec{R}_t^2 \rangle \sim t$ $(\gamma = 0)$ and ballistic motion $\langle \vec{R}_t^2 \rangle \sim t^2$ $(\gamma = \infty)$.

The 3D direction correlation function, also called cosine correlation function, for symmetric displacement pdfs with finite variance, is given by

$$C(t) = \langle \cos(\theta) \rangle \tag{4}$$

where the turn angles θ are taken between successive displacements to a time scale t. For a 3D motion the correlation function can be derived as the mean cosine of the turning angles c to the power of t [17],

$$C(t) = c^t. \tag{5}$$

We calculate the mean cosine of turning angles for their pdf, Eq. (2), as

$$c = \frac{\gamma^2}{1+\gamma^2}\coth(\pi\gamma/2), \tag{6}$$

where $\coth(x) \equiv (e^x + e^{-x})/(e^x - e^{-x})$. Finally, we readily obtain from Eq. (5) and Eq. (6) the 2d direction correlation function, given Eq. (2), as

$$C(t) = c^{t/2} = e^{-t/t_p}, \tag{7}$$

where

$$t_p = -2/\log(c) \tag{8}$$

is the persistence time. Note that Eq. (7) is independent of the spatial scale $1/\lambda$. Notably, for $\gamma = \infty$ the turn angle pdf becomes a delta function, and $C(t) = c = 1$ whereas the normal random walk case $\gamma = 0$ is represented in an uniform turn angle pdf implying a delta shaped correlation function $C(t) = \delta(t)$.

The 44 trypanosome trajectories display an exponential displacement distribution with mean value $\langle\delta\rangle = 1.26\mu m$. For the model we therefore assume the overall displacement distribution $P(\delta) = \lambda\exp(-\lambda\delta)$ with $\lambda = 1/\delta$. We plugged the fitted values for the persistence times t_p^{IW}, for intermediate walkers, and t_p^{PW}, for persistent walkers (Table 1 in the main manuscript), into Eq. (8). As explained in the main manuscript, the persistence time for the tumbling walker class t_p^{RW} is heavily determined by the fast rotation motion. Here we use, however, the fitted value $t_p^{\mathrm{RW}} \approx 0.60s$ for illustration. Finally, for the three motility modes, solving Eq. (8) for γ yields $\gamma_{\mathrm{RW}} = 1.21$, $\gamma_{\mathrm{IW}} = 6.55$, and $\gamma_{\mathrm{PW}} = 8.19$, respectively. Complementary to Fig.1 and Table I in the main text, we exhibit in Fig.1 below the turn angle distribution for each motility mode. The turn angle distribution for the tumbling walker class is already very close to true random walkers that would display a perfectly flat curve in Fig.1 (corresponding to the trivial value $t_p^{\mathrm{RW}} = 0$). Experimental trajectories were categorized using empirically found thresholds for the spread of the turn angle distribution.

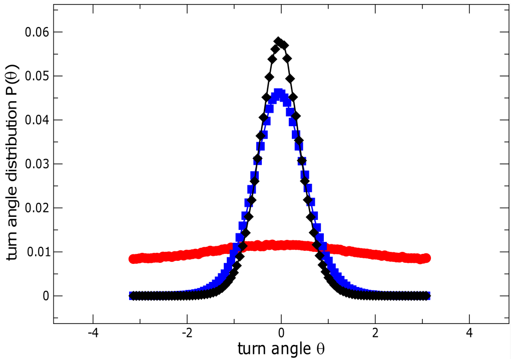

Figure S 1. Turn angle distributions for a short time lag ($\tau = 10$ simulation time steps ($t = 70s$)) for the three motility modes. Model parameters: $\lambda = 0.794$, $\gamma = 1.21$ (tumbling walkers, red), (b) $\gamma = 6.55$ (intermediate walkers, blue) (c) $\gamma = 8.19$ (persistent walker, black). Distributions averaged over 1000 realizations.
